# Computed Tomography in Infectious Endocarditis

**DOI:** 10.1016/j.jscai.2023.101292

**Published:** 2024-03-26

**Authors:** Eefje M. Dalebout, Alexander Hirsch, Jolanda Kluin, Tjebbe W. Galema, Jolien W. Roos-Hesselink, Ricardo P.J. Budde

**Affiliations:** a Department of Cardiology, Cardiovascular Institute, Thorax Center, Erasmus MC, Rotterdam, the Netherlands; b Department of Radiology and Nuclear Medicine, Erasmus MC, Rotterdam, the Netherlands; c Department of Cardiothoracic Surgery, Thorax Center, Erasmus MC, Rotterdam, the Netherlands

**Keywords:** cardiac imaging, computed tomography, infective endocarditis

## Abstract

Imaging is one of the cornerstones in diagnosis and management of infective endocarditis, underlined by recent guidelines. Echocardiography is the first-line imaging technique, however, computed tomography (CT) has a class I recommendation in native and prosthetic valve endocarditis to detect valvular lesions in case of possible endocarditis and to detect paravalvular and periprosthetic complications in case of inconclusive echocardiography. Echocardiography has a higher diagnostic accuracy than CT in detecting valvular lesions, but not for diagnosing paravalvular lesions where CT is superior. Additionally, CT is useful and recommended by guidelines to detect extracardiac manifestations of endocarditis and in planning surgical treatment including assessment of the coronary arteries. The advent of photon-counting CT and its improved spatial resolution and spectral imaging is expected to expand the role of CT in the diagnosis of infective endocarditis. In this review, we provide an overview of the current role of CT in infective endocarditis focusing on image acquisition, image reconstruction, interpretation, and diagnostic accuracy.

## Central Illustration

**Figure:**
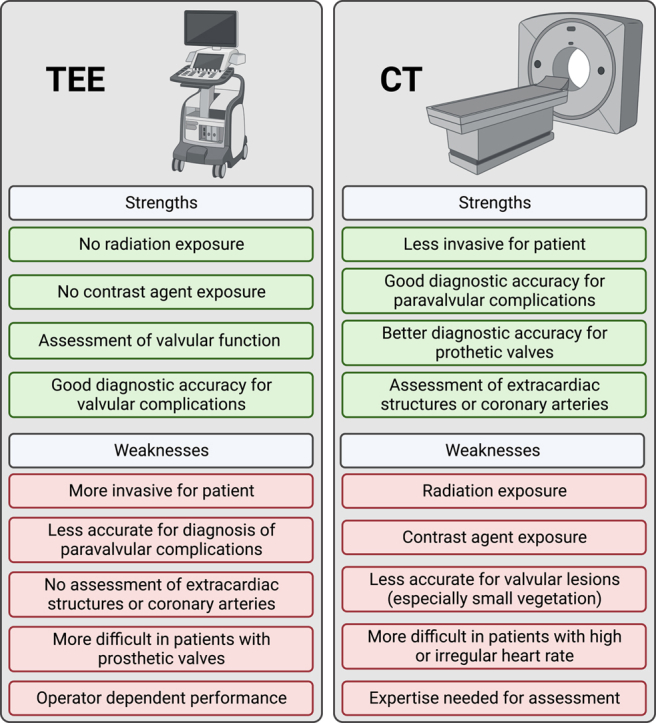

## Introduction

**Figure 1:**
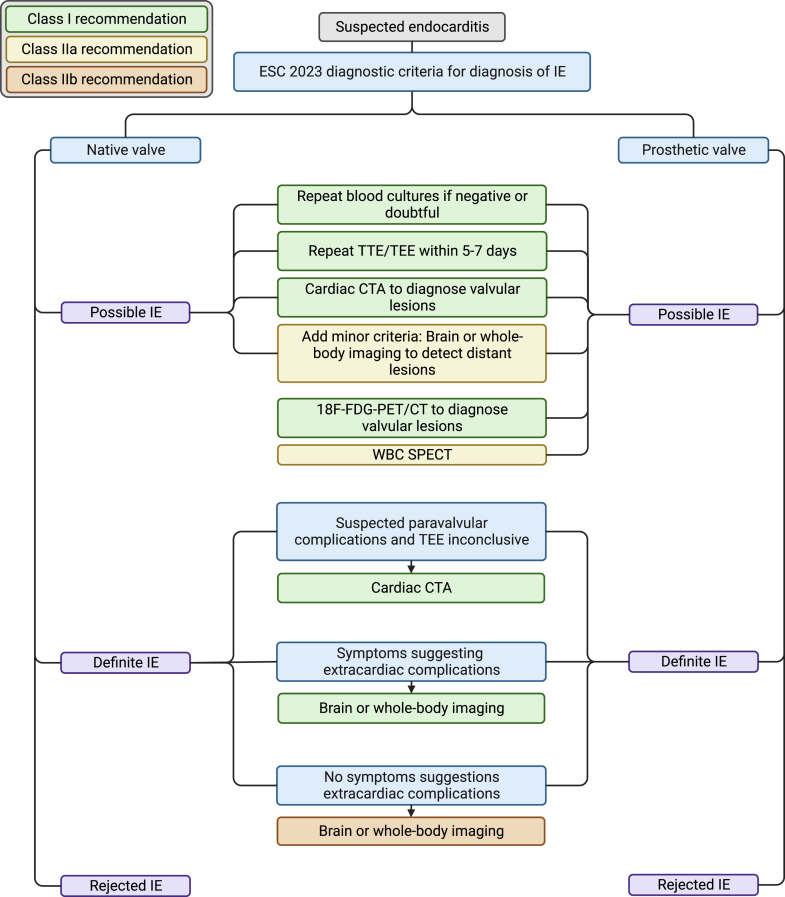
**The diagnostic pathway for patients with suspected infective endocarditis (IE) and the role of computed tomography based on the 2023 European Society of Cardiology (ESC) guidelines for the management of IE.****^2^** The color of the boxes indicates the class of recommendation. CTA, computed tomography angiography; TEE, transesophageal echocardiography; TTE, transthoracic echocardiography; WBC SPECT, white blood cell single photon emission computed tomography; 18F-FDG-PET/CT, 18F-fluorodeoxyglucose positron emission tomography and computed tomography.

**Figure 2:**
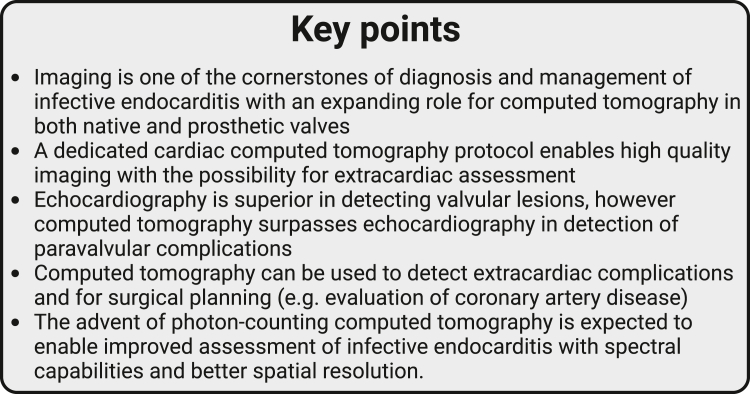
**Key points of this review**.

Diagnosis and management of infective endocarditis (IE) remains challenging, especially in patients with suspected prosthetic valve endocarditis. The estimated incidence of endocarditis is >1 million patients per year worldwide (estimated incidence rate 13.8 per 100,000 persons in 2019) and accounted for approximately 66,000 deaths worldwide.^1^ The incidence rate for endocarditis has increased over the past decades which is related to the increasing number of patients with implanted prosthetic valves and/or cardiac devices.^1^ Currently, the modified Duke criteria are used for diagnosis, consisting of 2 major and 5 minor diagnostic criteria.^2^^,^^3^ The 2 major criteria for diagnosis comprise positive blood cultures consistent with IE and signs of IE shown on imaging. Thus, imaging is one of the cornerstones of diagnosing IE. The standard cardiac imaging tool for IE is echocardiography, however, in the diagnosis of prosthetic valve endocarditis, the accuracy of echocardiography is diminished due to prosthetic shadowing.^4^ Furthermore, one of the minor criteria, the vascular phenomena, can be detected by whole body imaging techniques. The vascular phenomena consist of major distant or pulmonary emboli, infarcts and abscesses, mycotic aneurysms, intracranial ischemia or hemorrhage and osteoarticular septic complications (ie, spondylodiscitis).^2^^,^^3^ Over the past years, computed tomography (CT) has become increasingly important in the diagnosis and management of IE and is currently recommended to be used in both native and prosthetic valve endocarditis. This role of CT is supported by several guidelines and recommendations.^2^^,^^3^ The role of CT has recently been emphasized in the new 2023 European Society of Cardiology guidelines for the management of endocarditis with a class I recommendation for detecting valvular lesions in case of possible endocarditis and to detect paravalvular and periprosthetic complications in case of inconclusive echocardiography and when symptoms suggest extracardiac complications for suspected or definite native as well as prosthetic valve endocarditis (Figure 1).^2^^,^^3^^,^^5^, ^6^, ^7^ In case of suspected prosthetic valve endocarditis, CT has an important role, not only for evaluation of possible complications, but also because the diagnostic accuracy of echocardiography is limited.^4^^,^^8^, ^9^, ^10^ The aim of this review is to give an overview of the current role of CT in IE focusing on image acquisition, image reconstruction, interpretation, and diagnostic accuracy. The key points of this review are listed in Figure 2.

## CT protocol

### Patient preparation

For coronary CT angiography (CTA), β-blockers and nitroglycerine are often administered to reduce the heart rate and dilate the coronary arteries. The cardiac valves and aortic root can often be adequately assessed on scans acquired at higher heart rates and thus β-blockers and nitroglycerine are not required and may be contraindicated in patients with IE because of hemodynamic instability or severe valvular disease. When imaging of the coronary arteries is requested for surgical planning or detection of endocarditis-related complications of the coronary arteries, the risks, and advantages of using β-blockers and nitroglycerine should be carefully weighed on an individual patient basis.

### Contrast injection

Both enhanced and unenhanced images can be useful for evaluation of IE on CT. Standard coronary CTA contrast injection protocols often suffice for evaluation of left-sided IE. These protocols usually provide good contrast opacification of the left atrium and ventricle as well as the aorta and coronary arteries. Achieving homogeneous contrast enhancement of the right atrium and ventricle for assessment of right-sided IE is more challenging and multiphasic contrast injection or simultaneous injection in the arm and leg (that may also include mixed saline-contrast chasers) is advised to reduce blood-contrast mixing artefacts in the right atrium and ventricle and to obtain homogeneous contrast enhancement.^11^, ^12^, ^13^, ^14^ Also, delayed imaging may be helpful because of a more homogeneous distribution of contrast material in the body albeit with resultant lower contrast attenuation in the heart and large vessels. In case of severely reduced ventricular function or valve regurgitation, contrast arrival may be delayed and scan timing should be adapted accordingly.

Special caution in terms of the use and timing of contrast injection and scanning is needed in patients with congenital heart disease. Blood flow is often abnormal and surgical shunts can be present in patients with prior surgical repair and could thereby affect contrast medium distribution. A 2-phase contrast injection protocol has been proposed for imaging patients with congenital heart disease and showed promising results in a small cohort with different types of congenital heart disease.^14^ If contrast medium is suboptimal or inhomogeneously distributed, this could lead to inadequate visualization of cardiac chambers, vessels, or surgical shunts, and false-positive or false-negative findings. For example, in patients with Fontan circulation mixing of contrast circulating from the superior vena cava and blood entering from the inferior vena cava could lead to inhomogeneous contrast enhancement in the pulmonary artery, resembling pulmonary embolism, or vegetations.^15^ Simultaneous upper and lower limb injections of contrast can be used in patients with Fontan circulation and may solve this problem.^16^

### Image acquisition

A 3-phase image acquisition protocol provides a comprehensive cardiac assessment and is advised.

**Figure 3:**
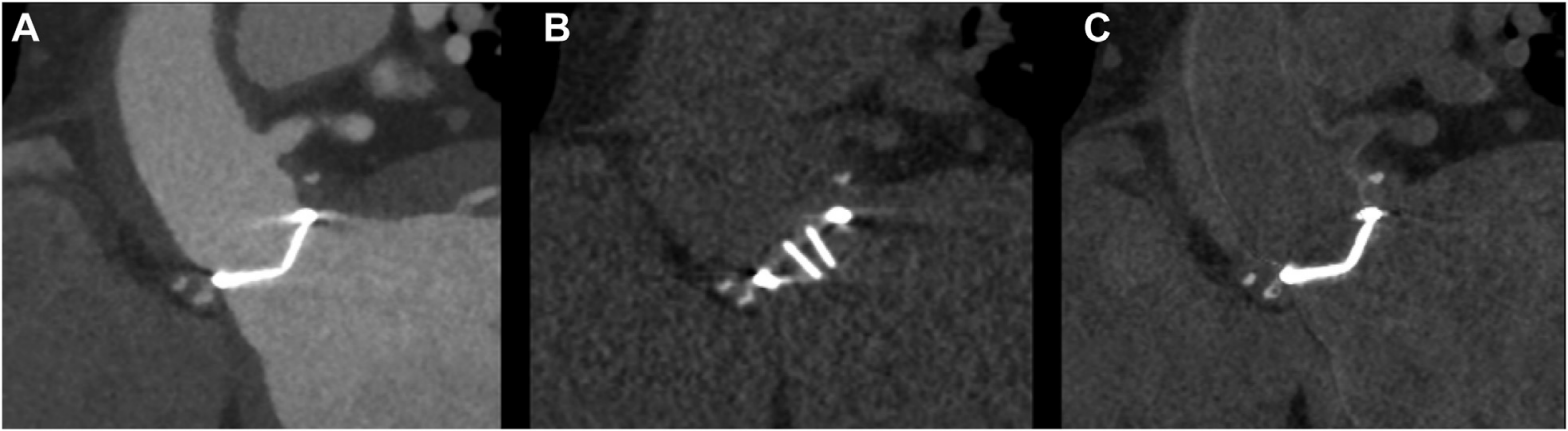
**Pledgets used in surgical aortic valve replacement shown on computed tomography in contrast-enhanced, true non-contrast, and virtual non-contrast images.** (**A**) Contrast-enhanced image, (**B**) true non-contrast image, (**C**) virtual non-contrast image.

First, a non-contrast enhanced acquisition is performed which is useful to evaluate calcifications and distinguish surgical material, such as suture pledgets, used during previous valve or aortic surgery, from paravalvular abscesses or dehiscence.^13^^,^^17^ In case of native valve endocarditis, the non-contrast enhanced scan may be omitted. When using a CT system with spectral capabilities the true non-contrast acquisition may be omitted as virtual non-contrast images can be reconstructed from the CTA (Figure 3).^18^

Secondly, an ECG-synchronized CTA including at least the heart (and in case of prior surgery preferably the entire ascending aorta and arch) is acquired. For dynamic evaluation of valve function, images reconstructed at least in diastole and systole are needed. Often it is easiest to set up the protocol in such a way that information on the full cardiac cycle is obtained (retrospective ECG-gating or wide-range prospective ECG-triggering).^10^^,^^19^, ^20^, ^21^, ^22^, ^23^ Although this is associated with radiation exposure, this is offset by the high morbidity and mortality rates associated with endocarditis.^9^^,^^24^ For dual-source CT systems, a dedicated prospectively ECG-triggered acquisition that covers the whole cardiac cycle and uses dose modulation has been described and provides good image quality with a relatively low radiation dose compared to retrospectively ECG-gated scans.^13^

Third, a delayed phase acquisition covering the entire chest (and in specific cases including the abdomen or brain as well) is acquired. This scan is used to assess the presence of extracardiac complications such as septic emboli or identify the probable cause of endocarditis (eg, spondylodiscitis). ECG-synchronization can be beneficial for this delayed phase but is not strictly necessary and a single phase is sufficient.

Most surgical prosthetic heart valves (PHV) cause only limited artifacts on CT.^25^^,^^26^ Further artifact reduction can be accomplished by higher tube voltage settings, even without increased radiation exposure, but results in less iodine contrast attenuation.^27^ When using CT platforms with dual-energy or spectral capabilities, iodine contrast can be maintained and optimized by reconstructing virtual monoenergetic image reconstructions at low keV. This technique can also significantly reduce metal artifacts caused by PHV by reconstructing images at high keV reconstructions.^28^^,^^29^ However, this comes at the expense of reduced temporal resolution in energy-integrating dual-source scanners but not with photon-counting CT (PCCT).^28^^,^^30^^,^^31^ Image reconstruction methods such as iterative reconstruction and metal artifact reduction reconstruction can further reduce PHV-related artifacts.^13^^,^^27^^,^^32^, ^33^, ^34^

### Image reconstruction

**Figure 4:**
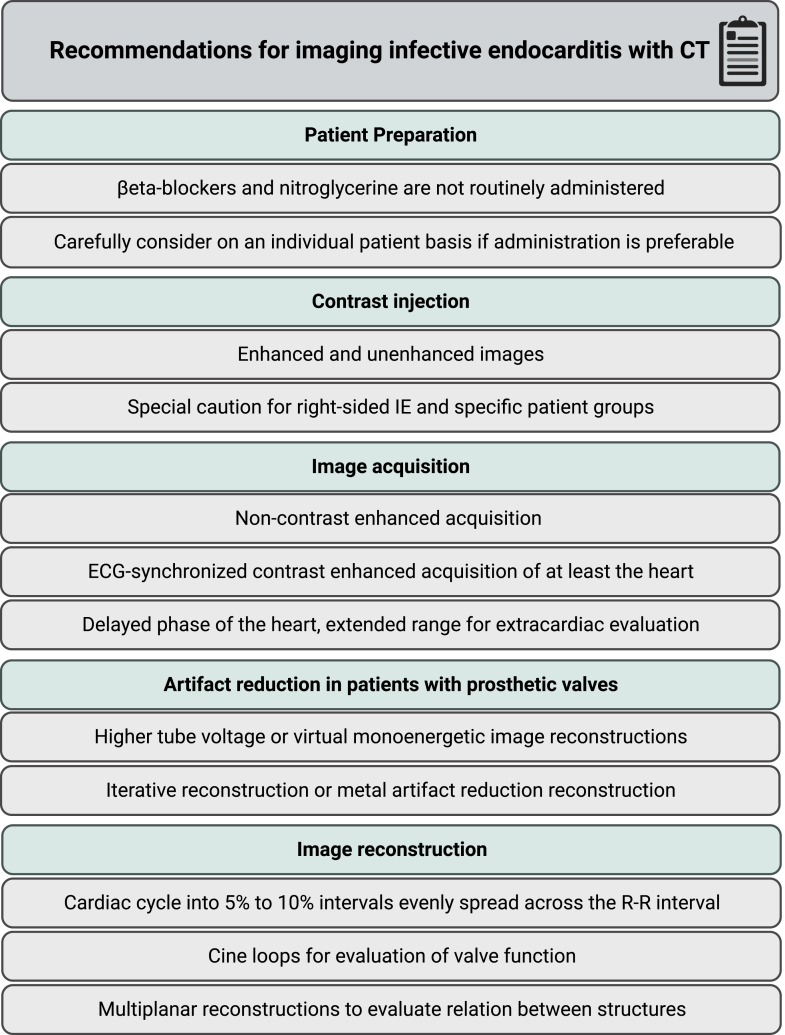
**Summary of computed tomography (CT) protocol recommendations for imaging infective endocarditis (IE).** ECG, electrocardiography.

CTA images should be reconstructed in 5% to 10% intervals evenly spread across the R-R interval. For the assessment of valves, reconstructions in plane, parallel, and perpendicular to the valve leaflets are reconstructed. Cine loops are used to detect a potential rocking valve, assess leaflet mobility, and calculate closing and opening angles for mechanical PHV.^35^ Habets et al provided an overview of normal mechanical PHV opening and closing angles based on manufacturer data.^36^ The standard echocardiographic views can be reproduced to correlate CT and echocardiography findings. When indicated, the coronary arteries can also be reconstructed in multiplanar reconstructions to demonstrate their relation to specific anatomical structures (eg, relation between the left main and an aortic root abscess or mycotic aneurysm). An overview of the recommendations on CT protocol in imaging IE is given in Figure 4.

## Findings in infective endocarditis

**Figure 5:**
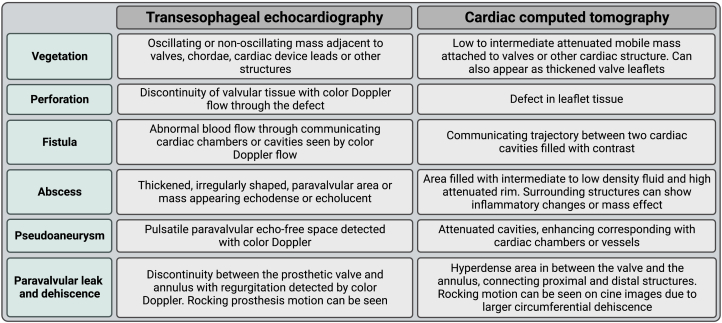
**Overview of signs of endocarditis that can be seen on echocardiography and computed tomography**.^8^^,^^10^^,^^37^, ^38^, ^39^

Vegetations, valve perforations, fistulae, paravalvular leakage or dehiscence, abscesses, and mycotic aneurysms are all signs of endocarditis that can be seen on CT. A summary of the characteristics and images of these abnormalities on both echocardiography and CT is provided in Figure 5.^8^^,^^10^^,^^37^, ^38^, ^39^

**Figure 6:**
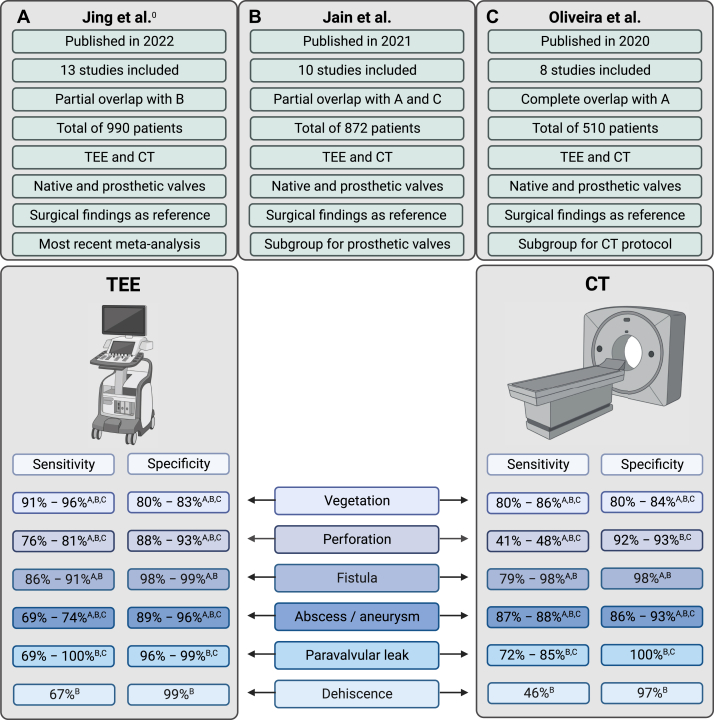
**Summaryof3 meta-analyses considering the diagnostic accuracy of transesophageal echocardiography(TEE)and computed tomography(CT)for infective endocarditis.** The most important study characteristics are provided and the range of pooled sensitivity and specificity for different signs of endocarditis. (**A**) Meta-analysis by Jing et al^40^; (**B**) meta-analysis by Jain et al^8^; (**C**) meta-analysis by Oliveira et al.^9^

Three meta-analyses that included a total of 510,872 and 990 patients (partially overlapping), including patients with native and PHV, have shown better diagnostic performance for echocardiography compared to CT for valvular lesions, while CT demonstrated a higher diagnostic accuracy for diagnosing paravalvular lesions.^8^^,^^9^^,^^40^ One meta-analysis performed subgroup analysis for multiphase (ie, multiple phases during the cardiac cycle) versus single-phase CT protocols and showed higher diagnostic accuracy in multiphase CT for pseudoaneurysm, vegetation, and paravalvular leakage detection compared to single-phase CT.^9^ The range of pooled sensitivity and specificity found in the 3 meta-analyses are indicated in Figure 6.^8^^,^^9^^,^^40^

### Vegetation

**Figure 7:**
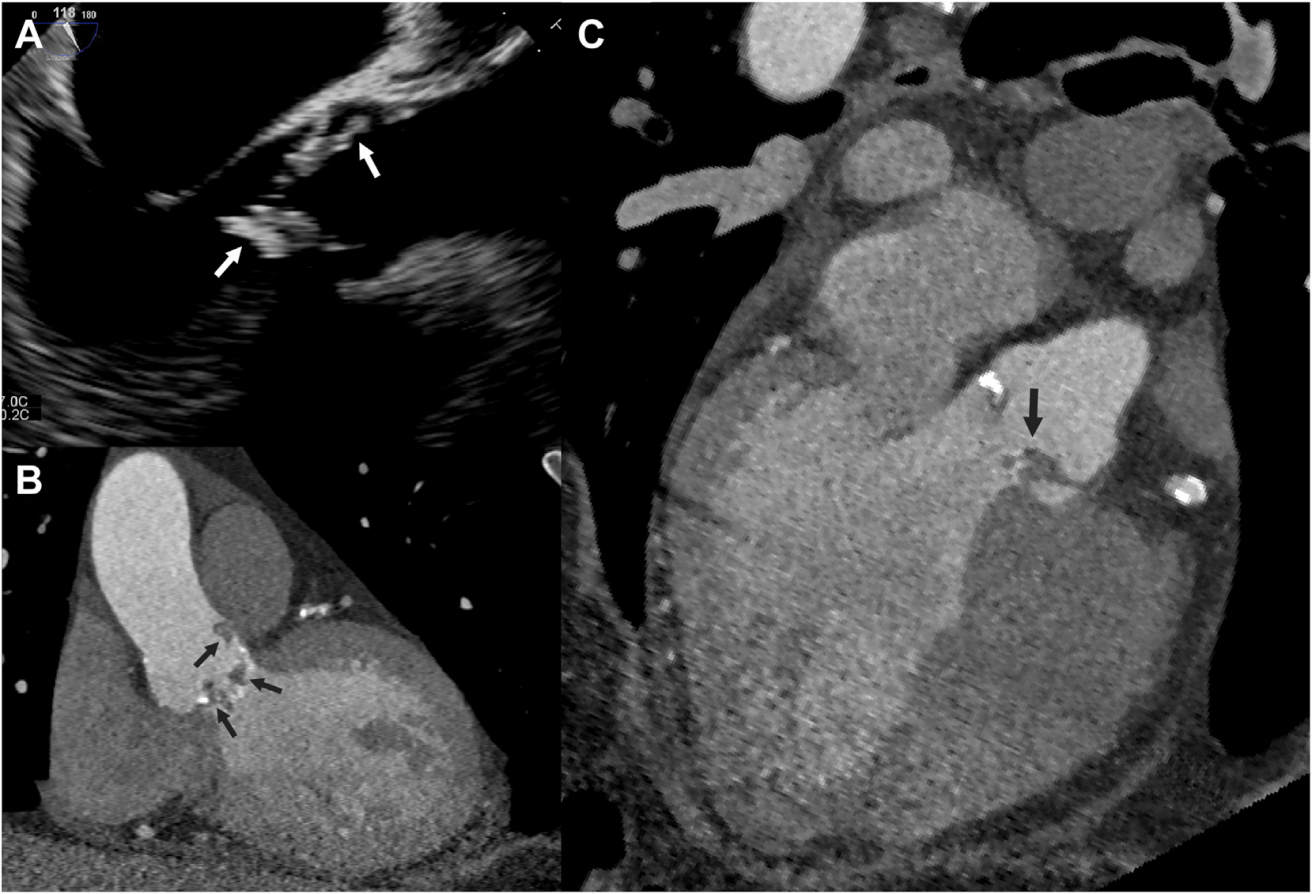
**Patient with a native aortic valve endocarditis and vegetations.** Vegetations (arrows) on the aortic valve and ascending aorta are seen on transesophageal echocardiography (**A**) and computed tomography (**B** and **C**).

**Figure 8:**
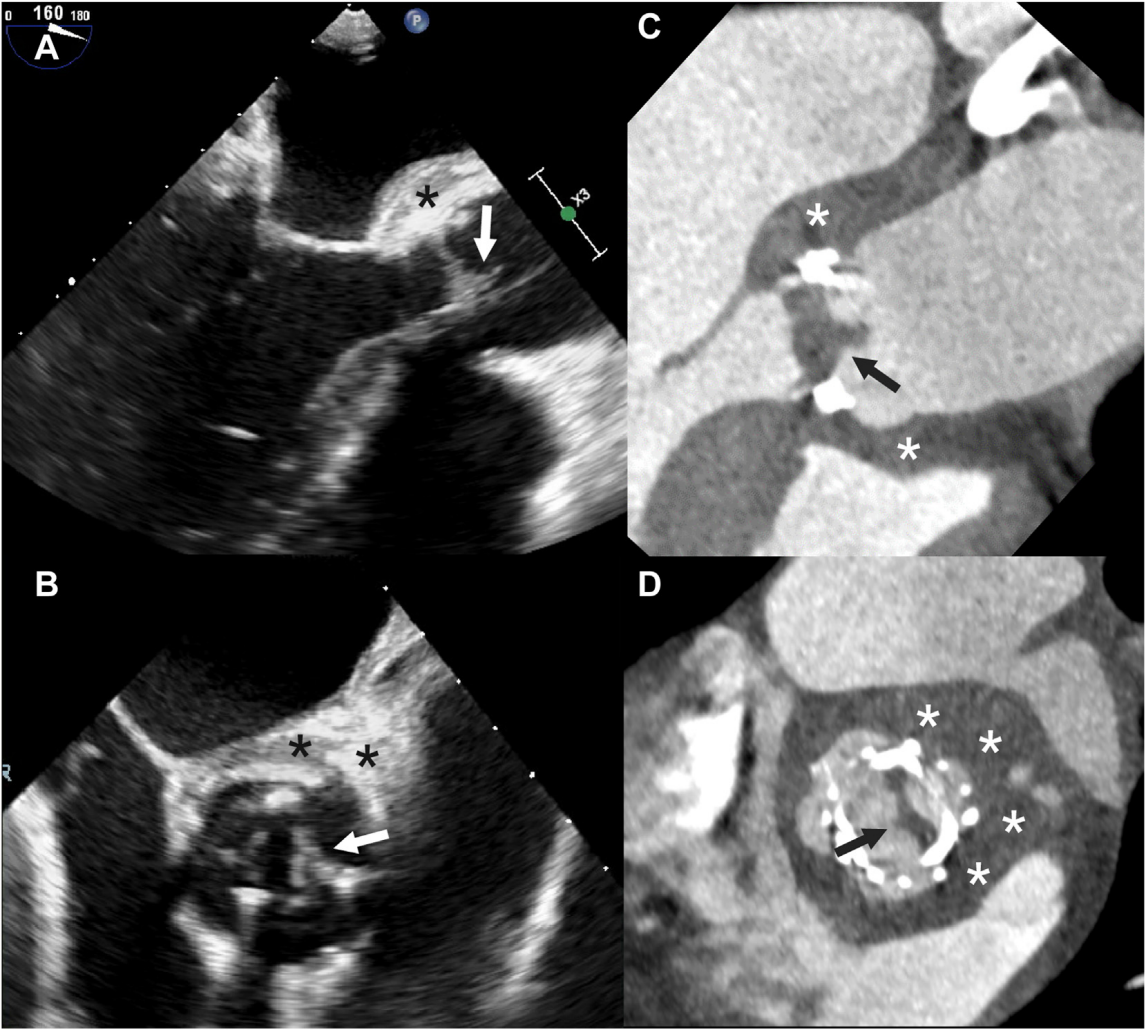
**Thickened valve leaflets and vegetations in a patient with aortic biological prosthetic heart valve endocarditis.** Thickened valve leaflets and vegetations on the valve are seen on both transesophageal echocardiography (**A** and **B**) and computed tomography (**C** and **D**). Also, notice the thickening of the aortic root (asterisks) indicating aortic root abscess formation.

Vegetations manifest as low to intermediate attenuation mobile masses, usually attached to valves or intracardiac prosthetic material or leads, but can also appear as thickened valve leaflets on CT (Figures 7 and 8 and Supplemental Videos 1-6).

The meta-analyses showed significantly higher pooled sensitivity and negative predictive value for transesophageal echocardiography (TEE) compared to CT for detecting vegetations.^8^^,^^9^^,^^40^ The pooled sensitivity ranges from 91% to 96% for TEE and 80% to 86% for CT and pooled specificity ranges from 80% to 83% in TEE and 80% to 84% for CT.^8^^,^^9^^,^^40^ One meta-analysis did a subgroup analysis for PHV and found significantly lower specificity for TEE (74%; 95% CI, 60%-84%) compared to CT (94%; 95% CI, 82%-98%) in detecting vegetations in PHV (*P* = .02).^8^

Regarding vegetation size, TEE and CT correlate poorly for small vegetations (<4 mm) and small vegetations were also often missed by CT assessment. But for larger vegetations (≥10 mm), TEE and CT correlate well.^41^^,^^42^ This cut-off is important because the risk of embolism and mortality increases with larger vegetations and the guidelines recommend early operative treatment in patients with large vegetation (≥10 mm) and signs of embolic events (symptomatic and asymptomatic).^2^^,^^3^^,^^42^^,^^43^

### Perforation

**Figure 9:**
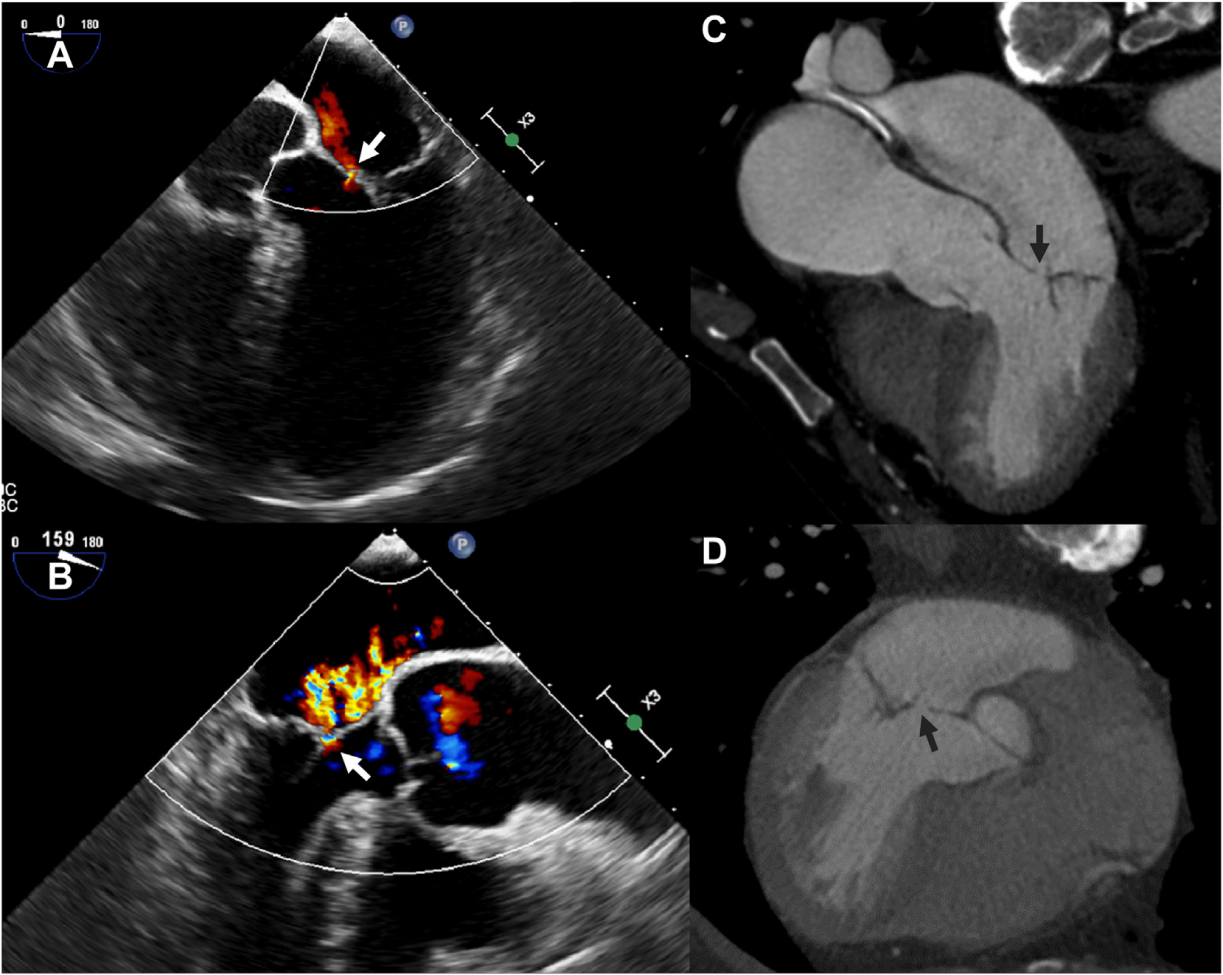
**Perforation of the anterior mitral valve leaflet in a patient with native mitral valve endocarditis.** The transesophageal echocardiography (**A** and **B**) images show the Doppler jet traversing the anterior mitral valve leaflet (arrow) indicating a perforation. The computed tomography images (**C** and **D**) show a focal discontinuity (arrow) in the valve leaflet compatible with a perforation.

On CT, perforation shows as a defect in the leaflet tissue and is often difficult to see (Figure 9 and Supplemental Videos 7-9). It is therefore not surprising that CT had a significantly lower sensitivity for detecting perforation than TEE.^8^^,^^9^^,^^40^ The pooled sensitivity for detecting perforation ranged from 76% to 81% for TEE and from 41% to 48% for CT. Specificity was not significantly different between TEE and CT.^8^^,^^9^^,^^40^

### Fistula

**Figure 10:**
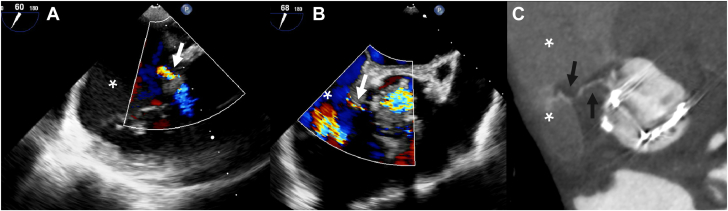
**Fistula between the aortic root and right atrium in a patient with aortic biological prosthetic heart valve endocarditis.** Fistula between the aortic root and right atrium (asterisks) shown on transesophageal echocardiography with a Doppler jet traversing from the aortic root to the right atrium (**A** and **B**) and computed tomography with a contrast-enhanced trajectory between the aortic root and right atrium (arrows) (**C**).

A fistula is seen as a communicating trajectory between 2 cardiac cavities (Figure 10 and Supplemental Videos 10-12). Two meta-analyses compared the diagnostic accuracy of TEE and CT for detecting fistulas. Pooled sensitivity ranged from 86% to 91% for TEE and 79% to 98% for CT. Specificity was comparable for TEE (98%-99%) and CT (both 98%).^8^^,^^40^

### Abscess and pseudoaneurysm

**Figure 11:**
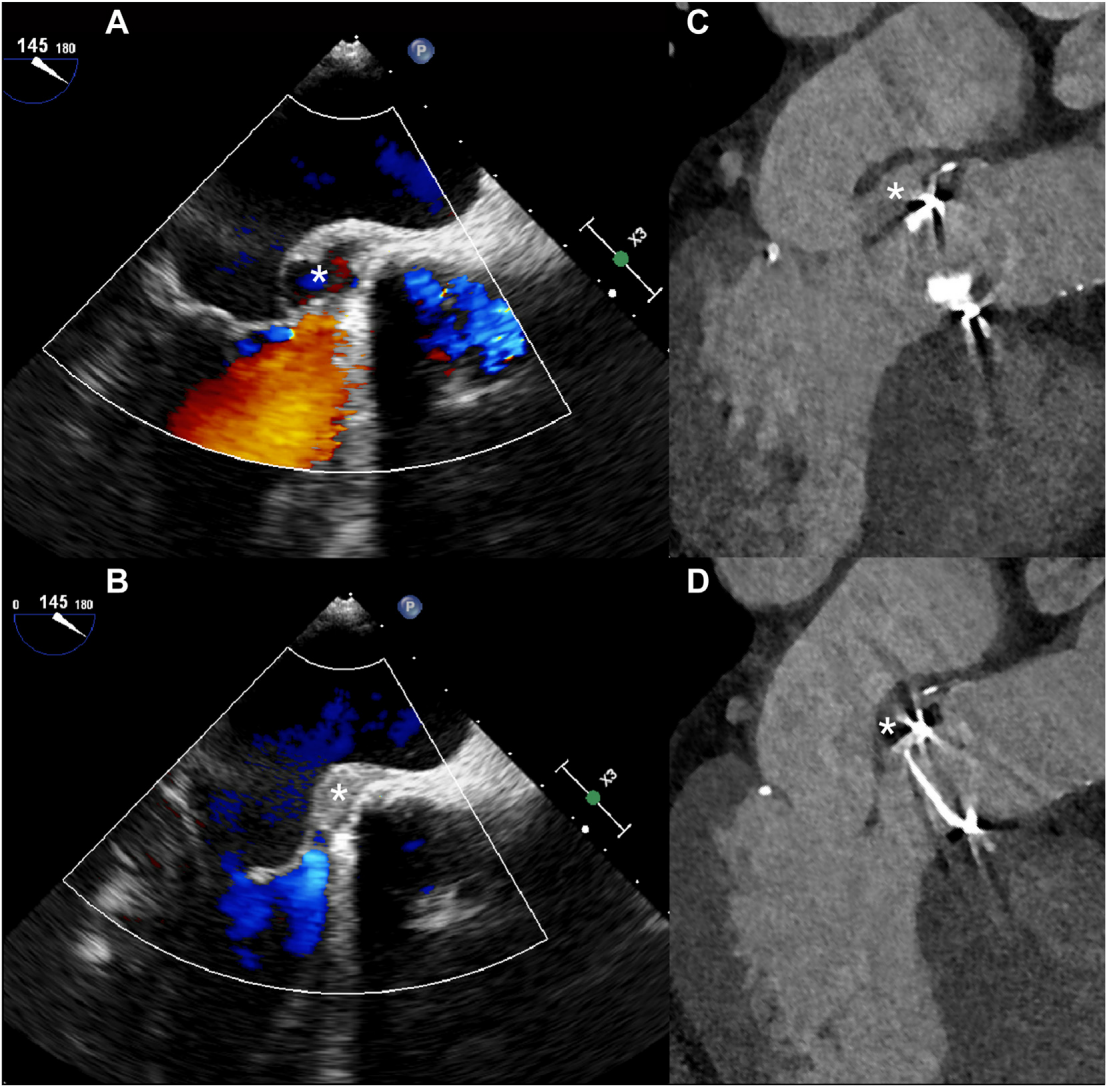
**Aneurysm between the aortic root and left atrium**. Aneurysm between the aortic root and left atrium filling with blood in the systolic phase and collapsing in the diastolic phase (asterisks) in a patient with endocarditis of a mechanical aortic valve shown on transesophageal echocardiography (**A** and **B**) and computed tomography (**C** and **D**).

Abscesses and pseudoaneurysms are often referred to as periannular complications. Classically, an abscess in the body is seen as a lesion filled with intermediate to low-density fluid and an enhancing rim on CT. However, aortic root abscesses in IE often are more difficult to discern on CT, especially in the arterial phase. A thickened and hypodense appearance of the aortic root tissue and fibrous continuity of the aortic valve and anterior mitral valve leaflet should raise suspicion of an abscess (Figure 8 and Supplemental Videos 3-6). Surrounding structures can show inflammatory changes or mass effect. Pseudoaneurysms on the other hand are easy to see on CTA as the cavity fills with contrast material and they are usually located close to the aortic valve. Cavity size can differ however throughout the cardiac cycle and thus the mycotic aneurysm may be more easily seen in specific phases of the cardiac cycle (Figure 11 and Supplemental Videos 13 and 14).

Sensitivity for detecting perivalvular complications is higher for CT compared to TEE.^8^^,^^9^^,^^40^ The pooled sensitivity ranged from 69% to 74% for TEE and 87% to 88% for CT. Pooled specificity was not significantly different and ranged from 89% to 96% and 86% to 93% for TEE and CT respectively.^8^^,^^9^^,^^40^ Jain et al found no significant differences in pooled sensitivity and specificity between TEE and CT for the detection of periannular complications in subgroup analyses for PHV.

The importance of detecting abscesses, pseudoaneurysms, and dehiscence and the role of imaging in detecting them in patients with IE is underlined by clinical guidelines. Both European and American guidelines for the diagnosis and management of IE recommend surgical intervention in patients with paravalvular complications.^2^^,^^3^

### Paravalvular leak and dehiscence

Paravalvular leak is seen as a hyperdense area in between the valve and the annulus, connecting proximal and distal structures and thereby depicting malalignment of the prosthesis. Rocking motion of the prosthesis can be seen on cine images if the prosthesis is dehiscent over a large part of its circumference.

Two of the meta-analyses described sensitivity for paravalvular leak. Oliveira et al found a sensitivity of 69% (95% CI, 58%-79%) for TEE and 72% (95% CI, 51%-88%) for multiphase CT.^9^ Jain et al found higher sensitivity of 100% (95% CI, 86%-100%) and 85% (95% CI, 67%-100%) respectively for TEE and CT.^8^ Specificity was high in both studies, ranging from 96% to 99% for TEE and both showed 100% specificity for multiphase CT.^8^^,^^9^

The meta-analysis performed by Jain et al was the only meta-analysis studying diagnostic accuracy for valve dehiscence. Sensitivity was 67% (95% CI, 54%-77%) for TEE and 46% (95% CI, 17%-78%) for CT. Specificity for TEE and CT was 99% (95% CI, 90%-100%) and 97% (95% CI, 92%-99%) respectively.^8^

### Extracardiac findings

**Figure 12:**
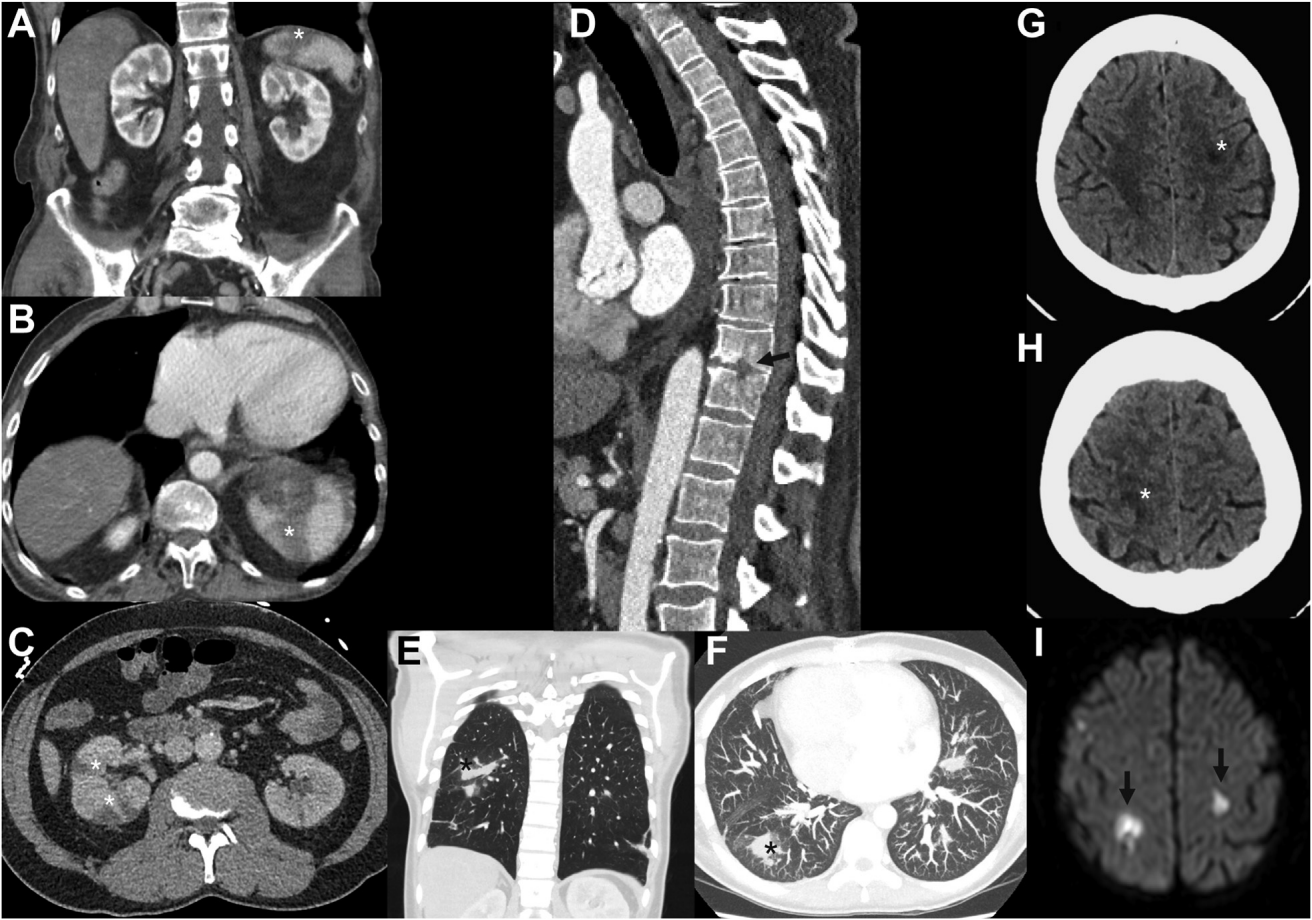
**Extracardiac complications shown on computed tomography.** Wedge-shaped hypoattenuating regions in the spleen and kidney (**A**, **B,** and **C**) are compatible with infarct due to embolized vegetations. Destruction of part of the vertebral bodies due to spondylodiscitis (**D**) and pulmonary septic emboli (**E** and **F**). Cerebral ischemia due to septic emboli seen on computed tomography (**G** and **H**) and magnetic resonance imaging (**I**).

Commonly seen extracardiac manifestations of IE are abscesses of the spleen or kidneys and septic emboli in for example the lungs, spleen, or brain, but emboli can be present in any organ (Figure 12).^44^ Septic emboli of the lung present usually as bilateral peripheral nodules with irregular walls and can show cavitary changes.^45^^,^^46^ Emboli of the spleen and kidneys are shown as 1 or multiple wedge-shaped hypodensities without enhanced rim.^45^^,^^47^ Abscesses are caused by the dissemination of bacteria from the valve or cardiac structures into the bloodstream causing metastatic infection in another organ. Infarction sites may become necrotic and progress into abscesses. Finally, the infectious site causing IE, for example, spondylodiscitis, other osteoarticular infections, or intestinal cancer, could be seen on CT.^45^

### Preoperative management

Coronary artery disease can also be evaluated using coronary CTA and can be used as an alternative to invasive angiography for preoperative evaluation of coronary artery disease.^48^ Coronary CTA has a high diagnostic accuracy for detecting coronary artery disease in the preoperative workup for elective valve surgery and is feasible in patients with a PHV.^49^, ^50^, ^51^ In patients with prior surgical coronary revascularization, CT can be used to assess bypass graft patency, course and the relationship between grafts and other structures in relation to the sternum. The aorta and femoral vessels can also be assessed and may provide important information for surgical planning.^52^ Moreover, CT can evaluate the location and course of mycotic aneurysms and abscesses and their relation and proximity to the coronary arteries and other cardiac structures, such as the left atrium, potentially carrying implications for surgical planning. A summary of the strengths and weaknesses of TEE and CT is given in the Central Illustration.

**Central Illustration:**
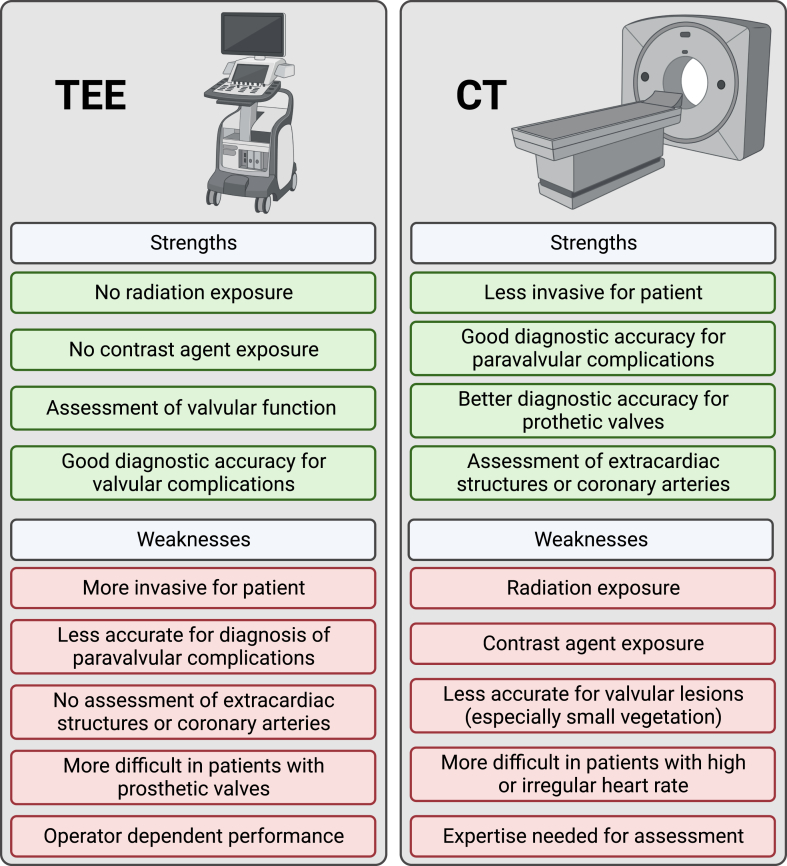
Overview of strengths and weaknesses of transesophageal echocardiography (TEE) and computed tomography (CT).

### Reporting

Systematic and complete interpretation and reporting of findings is important and consists of multiple aspects. Clinical context and abnormal findings of previous tests or scans should be known and mentioned.

Image quality and presence and extent of artifacts are described with special attention to artifacts that hamper the assessment of important structures. All heart valves should be examined for vegetations, perforations, leaflet thickening, and functioning. Prosthetic valve leaflet movement, opening and closing angles of the leaflets and paravalvular dehiscence should be assessed. Furthermore, mycotic aneurysms, abscesses and fistulas and their location, size and relation to surrounding anatomical structures are described. In patients with previous surgery or indication for surgery, coronary artery disease describing graft or stent location and patency, aortic diameters and calcification, the distance between the right ventricle and sternum and the presence of surgical material is important. Extracardiac findings, especially IE-related complications such as septic emboli, mycotic aneurysms and abscesses should be included as well. Finally, the findings should be related to the clinical context, previous scans or tests and if possible surgical reports and summarized in a concise and structured manner.

### Combined CT and nuclear imaging modalities

CT combined with nuclear imaging modalities allows for the integration of functional molecular imaging and anatomical information. Nuclear imaging modalities recommended for IE include white blood cell single photon emission computed tomography (WBC SPECT) and 18F-fluorodeoxyglucose positron emission tomography and computed tomography (18F-FDG-PET/CT).^2^^,^^3^ These nuclear imaging modalities are especially useful in prosthetic valve endocarditis and cardiac implantable electronic device infections. A meta-analysis on the use of these imaging modalities for IE found a pooled sensitivity of 81% (95% CI, 73%-86%) and a pooled specificity of 85% (95% CI, 78%-91%) for 18F-FDG-PET/CT with an area under the curve of 0.90. Pooled sensitivity of WBC SPECT was 86% (95% CI, 77%-92%) and pooled specificity of 85% (95% CI, 92%-99%) with an area under the curve of 0.96.^53^ Swart et al succeeded in further improving diagnostic accuracy for 18F-FDG-PET/CT in imaging prosthetic valve endocarditis by identifying and excluding several confounders, such as previous use of surgical adhesives.^54^ Finally, 18F-FDG-PET/CT and WBC SPECT also allow for the evaluation of extracardiac manifestations of IE such as septic emboli.^55^^,^^56^

## Future outlook

One of the most recent advances in CT imaging is the development of PCCT.^57^^,^^58^ The major advantages of PCCT are the improved spatial resolution and noise reduction as well as spectral imaging capabilities. Research on the benefits of PCCT in visualization of coronary artery disease has been done and shows better image quality with sharper borders of calcification and less blooming artifacts and thereby easier assessment of the degree of stenosis needing significantly lower radiation dose with PCCT compared to energy integrating detectors.^59^, ^60^, ^61^ Coronary stents are also sharper delineated with fewer artifacts and thus better evaluation of stent patency and in-stent stenosis.^62^ PCCT is also promising in reducing metal artifacts in surgical and transcatheter PHV. A case series in patients scanned after transcatheter aortic valve replacement showed detailed and sharply delineated stent frames and valve leaflets and function with limited artifacts.^63^ This could potentially lead to better assessment of PHV with more accurate diagnosis for patients with suspected prosthetic valve endocarditis. However, to our knowledge, there are no studies on PHV or diagnostic accuracy for IE-related signs using PCCT confirming this.

The spectral properties of PCCT allow for the reconstruction of virtual monoenergetic images and virtual non-contrast images and thereby improve contrast enhancement and lower radiation dose by omitting the need for a true non-contrast scan and lower doses of iodinated contrast material. However, this technique needs further optimization (Figure 3).^64^^,^^65^

The decrease in the extent of blooming artifacts is most likely due to higher spatial resolution in photon-counting detectors and can be further reduced with virtual monoenergetic images at higher keV.^66^^,^^67^ Therefore, coronary CTA should be considered in patients with IE and more extensive coronary artery disease and/or coronary stents over invasive coronary angiography, thereby omitting the need for invasive tests and combining evaluation of possible paravalvular lesions.

Finally, PCCT has a potential benefit in postoperative and follow-up scans in patients with PHV or vascular prostheses. Using virtual non-contrast scans, aneurysms, and endoleaks can be differentiated from calcifications in an aneurysm sack or surgical material such as pledgets.^58^^,^^68^

## Conclusion

Imaging remains one of the cornerstones of the diagnosis and management of IE. Although echocardiography is the first-line imaging tool, the role of CT is emerging in both native and prosthetic valve endocarditis and its use is strongly recommended in current guidelines. Echocardiography is better at detecting valvular lesions, however, CT surpasses echocardiography in detecting paravalvular lesions. A dedicated cardiac CT protocol should be performed to acquire good quality images for evaluation of IE and its complications and can be extended for evaluation of extracardiac complications and surgical planning such as coronary artery disease detection. Finally, future expectations hold expansion of the role of CT for evaluation of IE with the advent of PCCT.
