# Supplementary material for: Computed Tomography in Infectious Endocarditis
Source: J Soc Cardiovasc Angiogr Interv. 2024 Mar 26;3(3Part B):101292. doi: 10.1016/j.jscai.2023.101292 (PMC11308100; doi:10.1016/j.jscai.2023.101292)
Supplement: Supplementary Material [file mmc1.docx]

**Supplementary material**

**Supplemental videos for figure 7**

**Supplemental video 1**

Patient with a native aortic valve endocarditis and vegetations. The video shows vegetations on the aortic valve and ascending aorta on transesophageal echocardiography.

**Supplemental video 2**

Patient with a native aortic valve endocarditis and vegetations. The video shows vegetations on the aortic valve and ascending aorta on computed tomography.

**Supplemental videos for figure 8**

**Supplemental video 3**

Thickened valve leaflets and vegetations in a patient with an aortic biological prosthetic heart valve endocarditis. The video shows vegetations and thickened valve leaflets on the aortic valve on transesophageal echocardiography. Also notice the thickening of the aortic root indicating aortic root abscess formation.

**Supplemental video 4**

Thickened valve leaflets and vegetations in a patient with an aortic biological prosthetic heart valve endocarditis. The video shows vegetations and thickened valve leaflets on the aortic valve on transesophageal echocardiography. Also notice the thickening of the aortic root indicating aortic root abscess formation.

**Supplemental video 5**

Thickened valve leaflets and vegetations in a patient with an aortic biological prosthetic heart valve endocarditis. The video shows vegetations and thickened valve leaflets on the aortic valve on computed tomography. Also notice the thickening of the aortic root indicating aortic root abscess formation.

**Supplemental video 6**

Thickened valve leaflets and vegetations in a patient with an aortic biological prosthetic heart valve endocarditis. The video shows vegetations and thickened valve leaflets on the aortic valve on computed tomography. Also notice the thickening of the aortic root indicating aortic root abscess formation.

**Supplemental videos for figure 9**

**Supplemental video 7**

Perforation of the anterior mitral valve leaflet in a patient with native mitral valve endocarditis. The transesophageal echocardiography image shows a Doppler jet traversing the anterior mitral valve leaflet indicating a perforation.

**Supplemental video 8**

Perforation of the anterior mitral valve leaflet in a patient with native mitral valve endocarditis. The transesophageal echocardiography image shows a Doppler jet traversing the anterior mitral valve leaflet indicating a perforation.

**Supplemental video 9**

Perforation of the anterior mitral valve leaflet in a patient with native mitral valve endocarditis. The computed tomography image shows a focal discontinuity in the valve leaflet compatible with a perforation.

**Supplemental videos for figure 10**

**Supplemental video 10**

Fistula between the aortic root and right atrium in a patient with an aortic biological prosthetic heart valve endocarditis. A Doppler jet traversing the aortic root into the right atrium can be seen on transesophageal echocardiography.

**Supplemental video 11**

Fistula between the aortic root and right atrium in a patient with an aortic biological prosthetic heart valve endocarditis. A Doppler jet traversing the aortic root into the right atrium can be seen on transesophageal echocardiography.

**Supplemental video 12**

Fistula between the aortic root and right atrium in a patient with an aortic biological prosthetic heart valve endocarditis. Computed tomography shows a contrast enhanced trajectory between the aortic root and right atrium.

**Supplemental videos for figure 11**

**Supplemental video 13**

Aneurysm between the aortic root and left atrium filling with blood in systolic phase in and collapsing in diastolic phase in a patient with endocarditis of the mechanical aortic valve shown on transesophageal echocardiography.

**Supplemental video 14**

Aneurysm between the aortic root and left atrium filling with blood in systolic phase in and collapsing in diastolic phase in a patient with endocarditis of the mechanical aortic valve shown on computed tomography CINE images.
